# Differentially Expressed Proteins and Associated Histological and Disease Progression Changes in Cotyledon Tissue of a Resistant and Susceptible Genotype of *Brassica napus* Infected with *Sclerotinia sclerotiorum*

**DOI:** 10.1371/journal.pone.0065205

**Published:** 2013-06-11

**Authors:** Harsh Garg, Hua Li, Krishnapillai Sivasithamparam, Martin J. Barbetti

**Affiliations:** 1 School of Plant Biology, Faculty of Science, The University of Western Australia, Crawley, Western Australia, Australia; 2 The University of Western Australia Institute of Agriculture, Faculty of Science, The University of Western Australia, Crawley, Western Australia, Australia; Indian Institute of Science, India

## Abstract

Sclerotinia rot caused by *Sclerotinia sclerotiorum* is one of the most serious diseases of oilseed rape. To understand the resistance mechanisms in the *Brassica napus* to *S. sclerotiorum*, comparative disease progression, histological and proteomic studies were conducted of two *B. napus* genotypes (resistant cv. Charlton, susceptible cv. RQ001-02M2). At 72 and 96 h post inoculation (hpi), lesion size on cotyledons was significantly (*P*≤0.001) smaller in the resistant Charlton. Anatomical investigations revealed impeded fungal growth (at 24 hpi and onwards) and hyphal disintegration only on resistant Charlton. Temporal changes (12, 24, 48 and 72 hpi) in protein profile showed certain enzymes up-regulated only in resistant Charlton, such as those related to primary metabolic pathways, antioxidant defence, ethylene biosynthesis, pathogenesis related proteins, protein synthesis and protein folding, play a role in mediating defence responses against *S. sclerotiorum*. Similarly a eukaryotic translation initiation factor 5A enzyme with increased abundance in susceptible RQ001-02M2 and decreased levels in resistant Charlton has a role in increased susceptibility to this pathogen. This is the first time that the expression of these enzymes has been shown to be associated with mediating the defence response against *S. sclerotinia* in cotyledon tissue of a resistant cultivar of *B. napus* at a proteomics level. This study not only provides important new insights into the resistance mechanisms within *B. napus* against *S. sclerotiorum*, but opens the way for novel engineering of new *B. napus* varieties that over-express these key enzymes as a strategy to enhance resistance and better manage this devastating pathogen.

## Introduction

*Sclerotinia sclerotiorum*, the causal agent of Sclerotinia disease, is one of the most destructive and cosmopolitan of plant pathogens [1]. This necrotrophic fungal pathogen attacks over 400 plant species worldwide, and is now considered a serious threat to many economically important crops, including soybean (*Glycine max*), sunflower (*Helianthus annus*) and canola (*Brassica napus*) [2], [3]. The collective annual losses from *S. sclerotiorum* in the USA across different crop species exceeds $200 million [1], and yield losses as high as 24% have been recorded in canola in Australia [4]. Effective disease control measures against *S. sclerotiorum* continues to be a challenge because of the inefficiency of the chemical control in managing this disease, largely due to difficulty in timing the application with the release of ascospores [1]. Further, cultural practices tend to avoid or reduce the severity of Sclerotinia stem rot, but none effectively controls *S. sclerotiorum* on its own. Host resistance offers the only economic and sustainable method for effectively managing this disease. However, the level of host resistance to this pathogen is still inadequate [1], [5], except for few studies where useful levels of host resistance have been identified in *Brassica* spp. [5]–[7].

A complex combination of factors has been reported to determine the severity of the disease caused by *S. sclerotiorum* [8]. These include the ability of this pathogen to produce oxalic acid and various hydrolytic enzymes, such as pectinases and polygalactouronases, by which this fungus can establish itself within the host species so rapidly that it does not give adequate time for the host plant to fully engage defence responses [8]–[10]. A number of studies have thus focused on understanding the molecular aspects to pathogenicity of this fungus, with much emphasis given to oxalic acid and cell wall degrading enzymes [11]–[13] as well as on engineering/identifying resistance against various secretome of *S. sclerotiorum*. For example, Hu et al. [14] demonstrated that transgenic sunflower constitutively expressing a wheat oxalate oxidase gene exhibited enhanced resistance against this pathogen. Similarly, polygalacturonase inhibitor genes that responded to the infection caused by *S. sclerotiorum* have also been characterised in *B. napus* [15]. In addition, breeding efforts have been made to define the inheritance of resistance, mainly by identifying various quantitative traits loci (QTLs) associated with resistance against this pathogen [16], [17]. In spite of studies at the molecular level in relation to the various cell wall degrading enzymes, and of various breeding efforts to understand the genetic basis of resistance, defence responses of various host species against *S. sclerotiorum* have been, at best, poorly characterized. This may be a consequence of the multi-factorial defence responses that can occur in response to infection by this pathogen [18]. Hence, detailed molecular investigations are warranted to elucidate the mechanism of resistance against this pathogen. Identification of the genes mediating the defence responses against *S. sclerotiorum* will not only enhance the understanding of the molecular basis of resistance, but will also help to develop effective disease control measures and molecular markers for disease resistance [19].

Relatively few genomic-based approaches have been deployed so far which detail changes in gene expression profile mediating the host responses to the infection of *S. sclerotiorum*. For instance, Li et al. [10] identified several genes associated with fungal pathogenesis by monitoring expressed sequence tags (ESTs) generated from two cDNA libraries of fungal genes during mycelial growth of *S. sclerotiorum* in pectin medium or in infected tissues of *B. napus* stems. Subsequently, four studies based on microarray platform were conducted to investigate the *B. napus* responses to *S. sclerotiorum* [18], [20]–[22] and, one in soybean by using cDNA platform [19]. A quantitative RT-PCR approach has also been used by Yang et al. [23] to examine the expression of five orthologs of *B. napus* genes and by Eynck et al. [24] in *Camelina sativa* where monolignol biosynthesis was found to be associated in defence responses against *S. sclerotiorum*.

It is interesting that most of our knowledge of the molecular events occurring in the incompatible interaction of *B. napus*-*S. sclerotiorum* pathosystem has come from microarray analysis. However, there is no such study in which a proteomics approach has been deployed in the incompatible interaction of *B. napus-S. sclerotiorum* pathosystem, even though the protein profile of a compatible interaction of this pathosystem [25] and of fungal mycelia of *S. sclerotiorum* and its secretome have already been explored [26]. Proteomic analysis is now considered to be a powerful tool to study plant-pathogen interaction by which differentially expressed proteins induced in response to the pathogen challenge can be identified [27], [28]. This technique is a valuable complement for genomic approaches for investigations into plant-pathogen interactions at the molecular level, particularly as it provides a continuity between genome sequence information with the protein profile, which in turn indicates possible biochemical cellular pathways involved [29]. A poor correlation between the mRNA transcript levels and protein abundance reported in different studies further necessitates the use of such proteomics approaches [30], [31] in the *B. napus*-*S. sclerotiorum* pathosystem, in which the defence mechanism is poorly understood.

The discovery by Garg et al. [32] of a *B. napus* genotype (cv. Charlton) capable of resisting invasion by *S. sclerotiorum* at the cotyledon stage provided a model pathosystem to study the mechanism(s) of resistance to this pathogen. The present study reports for the first time resistance mechanisms at proteomics level in cotyledon tissue of a resistant *B. napus* host genotypes when challenged against *S. sclerotiorum*. Our findings are discussed within the context of change in abundance of proteins and associated morphological and histological changes in susceptible and resistant *B. napus* host genotypes in response to the pathogen challenge.

## Materials and Methods

### Host genotypes, S. sclerotiorum isolate and inoculation procedure

Two spring type *B. napus* genotypes, *viz.* Charlton and RQ001-02M2, were used in this study. The cv. Charlton has resistance to *S. sclerotiorum* while RQ001-02M2 is highly susceptible [32], [33]. Both genotypes were grown in 13.7×6.6×4.9 cm trays, each having eight cells and containing a soil-less compost mixture. Groups of four (eight cells) trays (two containing the resistant Charlton and two the susceptible RQ001-02M2) were placed randomly in one 10-L plastic storage box (34×13×23 cm), total 20 boxes. Two seeds of each genotype were sown in each cell and then thinned to one seedling per cell after emergence. Both test lines were grown under controlled environment growth room conditions of 18/14 (±1)°C (day/night), with light intensity of 250 μE m^–2^ s ^–1^. A highly virulent isolate of *S. sclerotiorum* (MBRS-5) collected from the Mount Barker region of Western Australia (WA), from a site where there was a significant disease, was used throughout in this study [33]. This isolate belongs to pathotype 76, the predominant *S. sclerotiorum* pathotype in Western Australia [34]. All the test conditions, inoculum storage, inoculum production, inoculation method and disease assessment were carried out as described by Garg et al. [32]. Inoculations were carried out when cotyledons were 10-d old. Macerated mycelial suspension at a concentration of 2×10^4^ fragments mL^−1^, prepared in sterilized liquid medium (Potato Dextrose Broth 24 g/L, Peptone 10 g/L, in water), was used. A total of four droplets of mycelial suspension of 10 μl were deposited on each seedling using a micropipette, with a single drop on each cotyledon lobe. The sterilized liquid medium (un-inoculated) was similarly deposited on the cotyledons of both lines as a control comparison. Disease progression was monitored at 24, 48, 72 and 96 h post inoculation (hpi).

### Histology

Cotyledons were sampled at 12, 24, 48 and 72 hpi. Six cotyledons from each treatment (inoculated and non-inoculated of both genotypes, and from separate inoculated boxes) were removed from each of the six plants at each time interval. Sampled cotyledons were decolourised by the acetic acid : ethanol : water (2:2:1) solution at 25°C. At the time of examination, cotyledons were washed with two changes of deionised water and stained with 1% cotton blue [35]. Whole wet mounts of cotyledons on microscope glass slides were then examined and photographed using a Zeiss Axioplan 2 microscope with an AxioCam digital photograph system with bright field optics [35]. Cotyledons were also sampled for anatomical studies at 24, 48 and 72 hpi. Three cotyledons (from three separate seedlings) from each treatment (inoculated and non-inoculated of both genotypes) were prepared for glycol methacrylate (GMA) biological tissue sampling as described by Hua Li et al. [36]. Cross sections were stained for detection of polyphenols and lignin (0.5% Toluidine Blue O in benzoate buffer, pH 4.4), and were examined and photographed using same microscope and digital photograph system as above.

### Protein extraction

Cotyledons were sampled at 12, 24, 48 and 72 hpi for this experiment. Twenty cotyledons at each time of sampling were randomly harvested from twenty different seedlings (per treatment), pooled and flash frozen in liquid nitrogen and then stored at −80°C until protein extractions were carried out. Experimental design comprised three replications (pooled cotyledon tissue from twenty different plants per replication), for each treatment (i.e. resistant, resistant control, susceptible and susceptible control), and for each time of sampling. Three independent protein extractions were performed (one protein extraction per replication) for each treatment and for each time of sampling.

Protein extractions were performed as described by Marra et al. [37], with some modifications. The pooled *B. napus* cotyledons (approximately 2 g/replication) were ground to a fine powder using liquid nitrogen and then suspended in 10 mL of cold (−20°C) acetone solution containing 20% trichloroacetic acid (TCA; Sigma-Aldrich, Australia) and 0.2% dithiothreitol (DTT; Sigma-Aldrich, Australia) in a centrifuge tube. The samples were maintained at −20°C for at least 4 h to allow complete protein precipitation, and then centrifuged (20 min, 30,000 g at 4°C). The supernatant was discarded and the pellet was re-suspended in 5 mL of cold acetone solution (−20°C) containing 0.2% DTT and centrifuged as described above. The dried pellet was re-suspended in a rehydration buffer containing 7 M urea, 2 M thiourea, 1% DTT, 2% 3-((3-cholamidopropyl)-dimethyl-ammonia)-1-propane sulfonate (CHAPS) (Sigma), 10 mM phenylmethylsulfonyl fluoride (PMSF) (Sigma). The samples were then centrifuged (60 min, 30,000 g at 20°C), and supernatant were recovered, and transferred to fresh eppendorf tubes and stored at –20°C. Protein concentration was determined by a Bradford Dc protein assay (Bio-Rad, Gladesville, NSW, Australia). The samples were then cleaned by using ReadyPrep^TM^ 2-D Cleanup Kit (Bio-Rad) according to the manufacturer's instructions in order to remove ionic impurities from the samples, re-suspended in rehydration buffer, and concentrations re-determined using the same protein assay, and finally, stored at –20°C until use.

### Two-dimensional electrophoresis (2-DE)

Isoelectric focusing (IEF) of protein extracts in the first dimension was mainly performed as described by Marra et al. [37] with some modifications. One 2-DE gel was performed for each replication for each treatment and for each time of sampling. IEF was performed by using 11 cm immobilized-pH-gradient (IPG) strips (Bio-Rad) with a pH range from 4 to 7. The strips were passively rehydrated overnight in a immobiline drystrip reswelling tray, with 500 μg of protein in 200 μl of solution containing 7 M urea, 2 M thiourea, 1% DTT, 2% CHAPS, 10 mM PMSF and 2% Bio-Lyte (Bio-Rad). IEF was performed using the PROTEAN IEF Cell system (Bio-Rad). IPG strips were focused at 300 V for 1 min, gradient from 300 to 3500 V for 1.5 h, and 3500 V for 4 h. The focused IPG strips were equilibrated in 10 mL of equilibration buffer containing 6 M urea, 50 mM Tris/HCl pH 8.8, 20% (v/v) glycerol, 2% (w/v) SDS, and 2% DTT for 10 min followed by a second equilibration in the same equilibration buffer containing 2.5% of iodoacetamide instead of DTT for another 10 min. IPG strips were finally loaded on a 12.5% polyacrylamide gels (20×20 cm, 1.5 mm thickness, containing 0.377 M Tris-HCl pH 8.8, 0.1% SDS, 0.5% ammonium persulphate, 12.5% acrylamide/bis, and 0.5% Tetramethylethylenediamine) in a PROTEAN II XI cell (Bio-Rad) along with precision wide range standard proteins (Sigma, USA) for molecular mass determination. Gels were run at 15 mA per gel for 30 min, and then increased to 30 mA per gel until dye front reached the bottom of the gel. Gels were fixed for three times in Colloidal Coomassie Blue (CCB) fixing solution (30% absolute alcohol and 2.0% of concentrated H_3_PO_4_) for 30 min each, rinsed three times in CCB rinsing solution (2.0% H_3_PO_4_) for 20 mins each, and then equilibrated in CCB equilibration solution (18% ethanol, 2% H_3_PO_4_, 15% (NH_4_)_2_SO_4_) for 30 min. Gels were finally stained with CCB equilibration solution containing 1% of Coomassie Brilliant Blue G-250 (Bio-Rad) for three days and then detained in distilled water until protein spots were clearly visible. Images of the 2-D gels were acquired by GS-800 imaging densitometer (Bio-Rad) with a red filter (wavelength 595–750 nm) and a resolution of 63.5×63.5 µm.

### Image analysis and protein identification

PDQuest software version 8.0.1 (Bio-Rad) was used to assemble the match sets where replicate gel groups were compared. Several stringent criteria were followed for the spots which were retained in each replicated group of the gels. For instance, every spot of the replicate group was matched manually, all the apparent artifacts were removed and any spots missed by the automated spot detection feature of the software were manually added. Finally, only those spots that were present in all the replicate of each treatment were retained in the replicate group of each treatment, such that the correlation coefficient for each individual replicate group was ∼1.0. The inbuilt Student's t-test module of the PDQuest software was used to analyze different replicate groups, and protein spots were identified that were significantly different (*P*<0.05) in response to the pathogen challenge in inoculated replicate groups of resistant or susceptible genotypes in comparison to the respective control (both qualitative and quantitative analyses were performed). Average spot intensities were measured from the filtered images (to reduce the ‘noise’) for each spot that was statistically measured as significantly different (by PDQuest software) for each replication across all the treatments and time courses. Each spot intensity value comprises the sum of the signal intensities (expressed as spot/optical intensity units) of all the pixels that make up the object. Additional Student's t-test comparisons was performed by using these spot intensities values of resistant or susceptible genotypes with respect to the control genotypes at a specific time point to verify the results of PDQuest software (e.g. resistant *vs* resistant control or susceptible *vs* susceptible control at 12, 24, 48 and 72 hpi, separately). Finally, only those protein spots that exhibited; a) statistically significant differences (*P*<0.05) and reproducible results in terms of their spot intensities measured through PDQuest software as well as by additional Student's t-test comparisons and, b) more than a 1.5-fold change in abundance were considered for further analysis. Expression ratios (fold changes) for each spot for every treatment across all the time points were calculated from the spot intensities data with respect to their control genotype (i.e. resistant *vs* resistant control or susceptible *vs* susceptible control at 12, 24, 48 and 72 hpi, separately).

Differentially expressed protein spots were excised with a sterile scalpel and excised pieces of the gel were further processed by Proteomics International, Crawely, Western Australia. Protein samples were trypsin-digested and peptides extracted using standard techniques as described by Bringans et al. [38]. Peptides were analysed by MALDI TOF/TOF (matrix-assisted laser desorption/ionisation time-of-flight) mass spectrometer using a 4800 Proteomics Analyzer (Applied Biosystems/MDS SCIEX). Briefly, the dry peptide solution was reconstituted in 2 ml standard diluents (30∶70 Acetonitrile:water) and the resulting solution was further diluted in 1∶10 ratio with matrix solution (a-Cyano-4-hydroxycinnamic acid, 10 mg/ml) [38]. Samples were spotted on 384-well Opti-TOF stainless steel plate and were analysed using a first run of standard TOF MS. A second run of MS/MS was focused on the 15 most intensive peaks of the first MS (excluding peaks known to be trypsin) with a laser set to fire 400 times per spot in MS mode and 2000 times per spot in MS/MS mode [38].

Mass spectra were analysed to identify protein(s) of interest using Mascot sequence matching software (Matrix Science Ltd., UK) with Ludwig NR Database (http://www.matrixscience.com/help/seq_db_setup_nr.html). The protein spots were identified as being ‘significant hit’ (P<0.05) based on individual peptide ion score. These peptide ion scores is automatically calculated by Mascot programme as -10*Log(P), where P is the probability that the observed match is a random event. When the individual ion score exceeds the threshold value for a random event, it indicates sequence identity or extensive homology (P<0.05) (Matrix Science Ltd., UK) (Table S1). Normally the identity of the spot is established as the protein that produced the highest score and consequently, the best match with its peptide sequence [39]. No species restrictions were applied while performing the searches across the database as the *B. napus* genome is not yet available.

## Results

### Disease progressions

**Figure 1:**
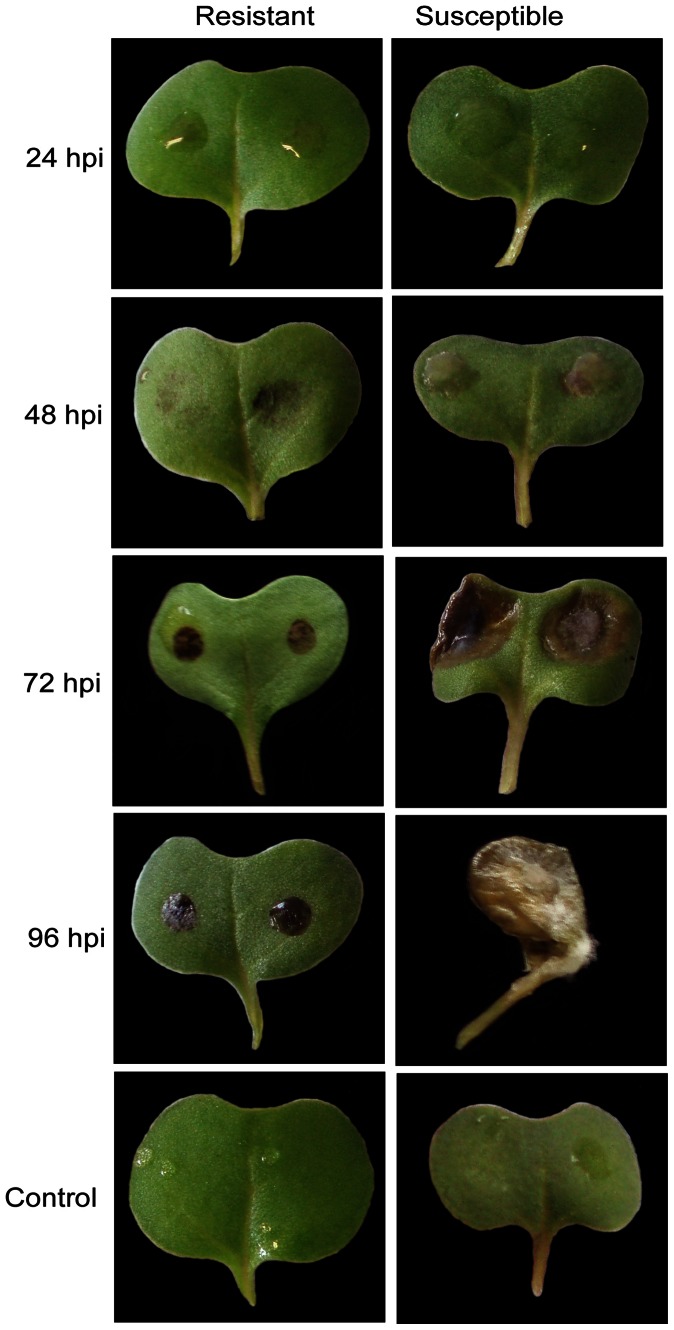
Appearance of *Brassica napus* resistant Charlton and susceptible RQ001-02M2 when inoculated with *Sclerotinia sclerotiorum.* Samples were taken at 24, 48, 72, 96 hours post inoculation (hpi). “Control” represents the mock inoculated control comparison for resistant and susceptible genotypes.

The responses of the resistant and susceptible genotypes following inoculation and their respective disease progressions at 24, 48, 72 and 96 hpi are shown in Figure 1/Figure S1. Cotyledon lesion diameters at 72 and 96 hpi were significantly greater (*P*≤0.05) in the susceptible RQ001-02M2 as compared with resistant Charlton. Water-soaked lesions were visible on cotyledons of the susceptible genotype at 48 hpi. After 48 hpi, an increase in cotyledon lesion diameter was observed only on the susceptible genotype with its mean value progressing from 3.4 mm at 48 hpi to 6.2 mm at 72 hpi, and then to 10.5 mm at 96 hpi. Furthermore, cotyledons of the susceptible genotype were covered with white mycelial growth by 96 hpi. In contrast, lesions on the resistant genotype remained small (approx. 3.5 mm) and were always confined within the diameter of the inoculum droplet at 48, 72 and 96 hpi.

### Histological differences

12 hpi: There were no differences between the resistant Charlton and the susceptible RQ001-02M2 in relation to the hyphal growth on the cotyledon surface at 12 hpi (Table 1).

**Table 1:** Description of growth of *Sclerotinia sclerotiorum* isolate MBRS-5 on the cotyledon surface of resistant (*Brassica napus* Charlton) and susceptible (*B. napus* RQ001-02M2) genotypes over time (12 to 72 hours post inoculation).

Hours post inoculation	Resistant Charlton	Susceptible RQ001-02M2
12	No increase in hyphal length	No increase in hyphal length
24	Significantly impeded hyphal growth as compared to susceptible genotype, increase in hyphal cell diameter	Extensive hyphal growth, but confined within the inoculum droplet area
48	Significantly impeded hyphal growth, increase in hyphal cell diameter	Extensive hyphal growth, hyphae extended beyond the periphery of the inoculum droplet area
72	Hyphal growth within the confines of the inoculum droplet area and/or disintegration of hyphal cell wall	Whole cotyledon covered with mycelial growth

**Figure 2:**
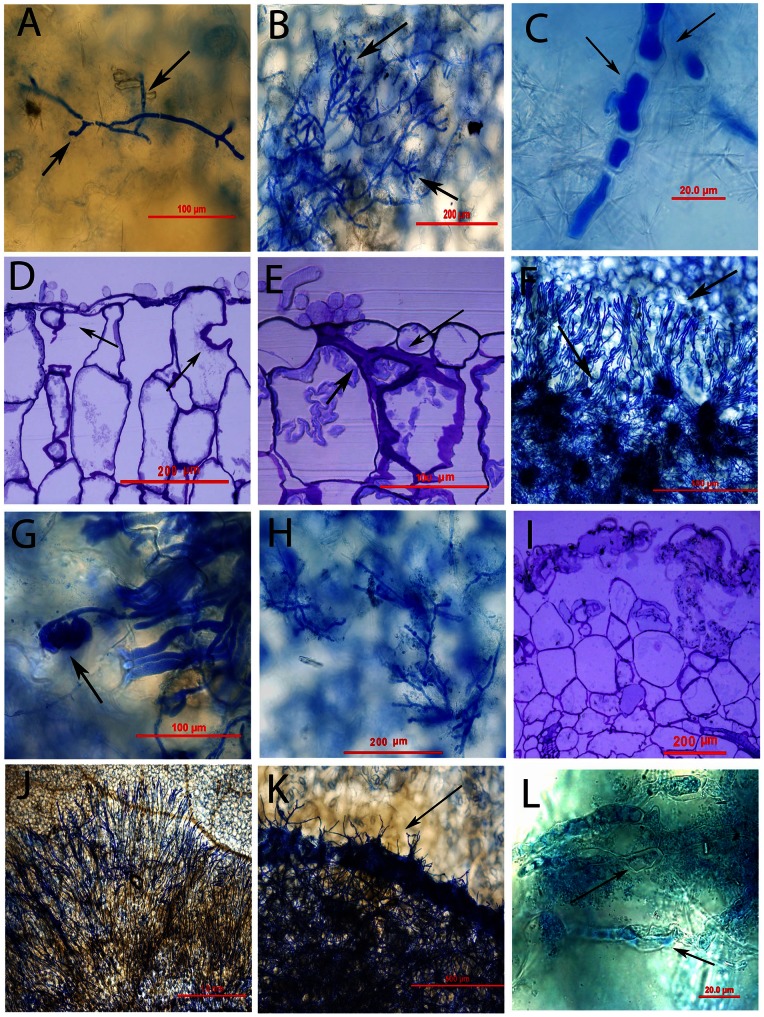
Histology of resistant and susceptible *Brassica napus* genotypes in response to *Sclerotinia sclerotiorum*. Spring type *B. napus* resistant Charlton and susceptible RQ001-02M2 were inoculated with *S. sclerotiorum* isolate MBRS-5. (A)–(C), (F)–(H), (J)–(L) Samples were cleared in acetic acid: ethanol: water (2∶2∶1), stained with 1% cotton blue, and photographed using a Zeiss Axioplan 2 microscope photograph system. (D)**,** (E), (I) 2 μm thick sections obtained and photographed using the same photograph system. (A) Impeded fungal growth on resistant Charlton at 24 hours post inoculation (hpi). Arrow indicates the presence of simple appresoria. (B) Hyphal growth on susceptible RQ001-02M2 at 24 hpi. Arrows indicate the presence of simple appresoria. (C) Increase in hyphal diameter of fungal cells on resistant Charlton at 24 hpi. (D) Cytoplasmic disorganization and necrotic cells (arrows) of palisade mesophyll cells in the susceptible RQ001-02M2 at 24 hpi. (E) Darkly-stained areas (arrows) around the dead cells of palisade mesophyll layer at 24 hpi in the resistant Charlton. (F) Hyphal growth on cotyledons of the susceptible RQ001-02M2. Arrows indicate the extension of hyphal growth beyond the periphery of the inoculum droplet area. (G) Repeated dichotomous branching of the terminal hyphae led to formation of appresoria (arrow) at 48 hpi on susceptible RQ001-02M2. (H) Hyphal growth on resistant Charlton at 48 hpi. (I) Fungal invasion up to palisade mesophyll cells and extensively damaged upper epidermis in the susceptible RQ001-02M2 at 48 hpi. (J) Hyphal growth on susceptible RQ001-02M2 extended across almost whole of the upper surface of the cotyledon at 72 hpi (K) Hyphal growth within the periphery of the inoculum droplet area (arrow) on resistant Charlton at 72 hpi. (L) Disintegration of hyphal cell wall (arrows) on resistant Charlton at 72 hpi.

24 hpi: Hyphae continued to grow on the cotyledons of both resistant Charlton and susceptible RQ001-02M2 by 24 hpi. However, hyphal growth on the resistant Charlton (Figure 2A) was significantly (*P*<0.001) impeded as compared to the susceptible RQ001-02M2 (Figure 2B; Table 1). The dichotomous branching of the terminal hyphae was apparent both on resistant Charlton and susceptible RQ001-02M2, resulting in the formation of simple appresoria (Figure 2A, 2B). There was, however, an increase in the diameter of hyphal cells on resistant Charlton (Figure 2C). Anatomical examinations of the resistant Charlton and the susceptible RQ001-02M2 showed cytoplasmic disorganization of the palisade mesophyll cells underneath of the intact upper epidermis layer (Figure 2D). Furthermore, toluidine blue-stained sections of the resistant Charlton showed a number of darker blue-stained regions around the dead cells of the palisade mesophyll layer (Figure 2E), which were not evident on the susceptible RQ001-02M2.

48 hpi: On the cotyledons of susceptible RQ001-02M2, extensive hyphal growth with the appearance as a mycelial mat was observed but only within the confines of the inoculum droplet area, some strands of hyphae had also extended beyond the periphery of the inoculum droplet area (Figure 2F), and the dichotomous branching of the terminal hyphae led to the formation of complex appresoria (Figure 2G). In contrast, hyphal growth on resistant Charlton was significantly impeded (*P*<0.001) (Figure 2H), a trend similarly observed at 24 hpi. Anatomical studies of the susceptible RQ001-02M2 revealed extensively damaged upper epidermis and palisade mesophyll cells with hyphal invasion up to the spongy mesophyll layer (Figure 2I). In contrast, hyphal invasion was mainly confined to the upper epidermis in the resistant Charlton with extensive disorganization of palisade mesophyll cells observed underneath of an intact epidermis layer.

72 hpi: In the susceptible RQ001-02M2, hyphae emerging from the inoculum droplet area extended across almost whole of the upper surface of the cotyledon (Figure 2J). Approx. 30% of the inoculated samples of the resistant Charlton were also observed with extensive mycelial growth on the cotyledon surface, which, however, seemed to be within the periphery of the inoculum droplet area, with limited strands of hyphae emerging beyond it (Figure 2K). An increase in diameter of hyphal cells was also apparent in the resistant Charlton followed by the disintegration of the hyphal cell wall (Figure 2L). The mycelial mat within the inoculum droplet area on resistant Charlton appeared darker (Figure 2K) compared with the susceptible RQ001-02M2.

#### Differential proteins from the interaction between S. sclerotiorum and the two *B. napus* genotypes

**Figure 3:**
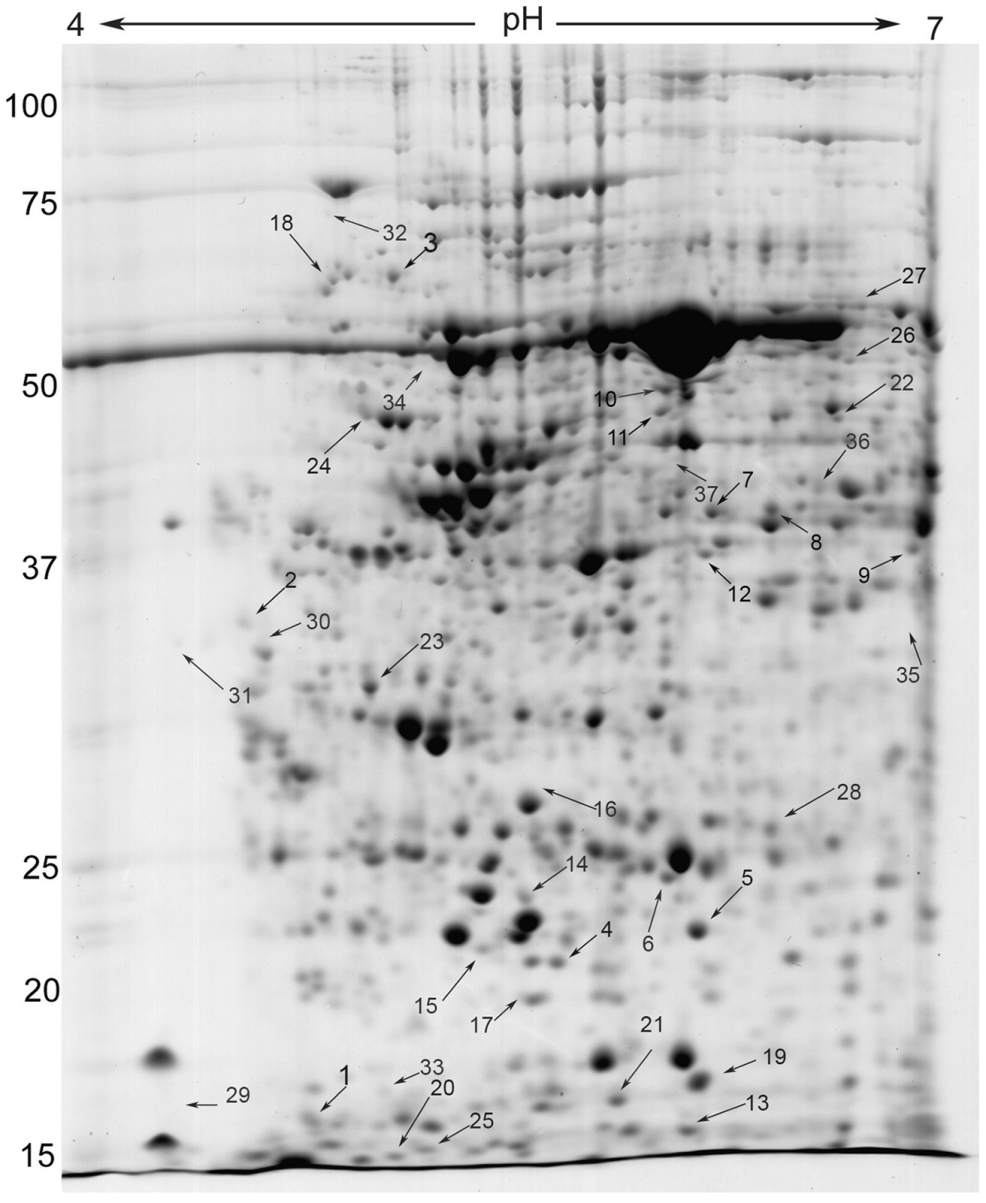
Representative image of resistant cv. Charlton cotyledon proteins separated by two-dimensional electrophoresis (2-DE). The 2-DE was performed for both *Brassica napus* resistant Charlton and susceptible RQ001-02M2 at 12, 24, 48 and 72 hours post inoculation (hpi) by using 11 cm immobilized-pH-gradient (IPG) strips. Gels were stained with Coomassie Brilliant Blue (CCB) and their images were acquired by GS-800 imaging densitometer (Bio-Rad) with a red filter (wavelength 595–750 nm) and a resolution of 63.5×63.5 µm. The numbers shown correspond with the spot numbers mentioned in Table 2.

Comparative proteome analysis of the resistant Charlton and the susceptible RQ001-02M2 in response to the *S. sclerotiorum* infection was conducted at 12, 24, 48 and 72 hpi and a representative image of a 2-DE gel is shown in Figure 3. An average of 400 protein spots in the resistant Charlton and 380 in the susceptible RQ001-02M2 were detected that were resolved within the pH range of 4–7 across the different time points. A total of 55 protein spots were identified as differentially regulated in the resistant Charlton and/or the susceptible RQ001-02M2. Out of these 55 protein spots, 37 spots were identified through ESI-Q-TOF-MS/MS analysis as being ‘significant hit’ (P<0.05) based on individual peptide ion score (Table S1). Among these 37 spots, only 8 spots could be matched to *Brassica* sp. as complete genome sequence of *B. napus* is not yet available. Identities of twelve protein spots were matched to *Arabidopsis thaliana*, two were from the Brassicaceae family viz. *Raphanus sativus* and *Capsella rubella* and, nine spots were of pathogen origin that were most likely extracted from the infected tissues of either/both *B. napus* genotypes. The change in abundance of individual proteins in each treatment across the different time points is shown in Table 2. Details of the Mascot analysis of the differential proteins identified in *B. napus* resistant Charlton and susceptible RQ001-02M2 are shown in Table S1. Of the 28 spots that were identified as of plant origin, 17 spots at 72 hpi, 16 at 48 hpi, 9 at 24 hpi and 19 at 12 hpi time points were identified. Twenty one of the 28 protein spots were significantly affected in response to the pathogen challenge across more than one time point (e.g. spot 3,4,5,6; Table 2). Qualitative differences between resistant and susceptible genotypes were also observed for spots 6, 9 and 16 as they were solely identified in either of the genotype (as a consequence of an absence of particular protein spots in the same relative positions on the 2-DE gels). Eleven protein spots (spots 6,7,8,9,10,11,12,13,19,24,28; Table 2) were found to be up-regulated only in resistant Charlton. For a few protein spots, the intensities were either significantly increased or decreased in both the resistant Charlton and the susceptible RQ001-02M2 (e.g. spots 3 and 22). A few protein spots were also identified with significantly increased intensity in the susceptible RQ001-02M2 but decreased in intensity in the resistant Charlton or *vice versa* (e.g. spots 1, 8 and 21). Further, three proteins identified in this study were detected at more than one position in the 2-DE gels (spots 7 and 8, 22 and 26, 17 and 25). The expression ratio calculated for each spot for every treatment across every time point clearly sets out the modulation of the proteins in response to the pathogen challenge across different time points (Table 2). Closer views of the gels images of each proteins spots with significant change in abundance are shown in Figures S2 and S3.

**Table 2:** Details of the proteins identified in *Brassica napus* resistant Charlton and susceptible RQ001-02M2 at various times after inoculation with *Sclerotinia sclerotiorum.*

Spot no^A^	Protein name	hpi^B^	Resistant		Susceptible	
			Expression^C^ ratio	SE^D^	Expression^C^ ratio	SE^D^
1	50S ribosomal protein L12-C [*Arabidopsis* thaliana]	72	−4.9	±0.9	+4.4	±0.1
2	Protein grpE [*Prochlorococcus marinus*]	48	*	*	−1.5	±0.0
3	Protein disulfide isomerase [*Brassica carinata*]	12	+1.8	±0.2	*	*
		48	*	*	+2.1	±0.3
		72	+2.4	±0.3	+2.5	±0.3
4	Light-harvesting complex I chlorophyll a/b binding protein 1 [*Arabidopsis thaliana*]	12	+3.1	±0.2	*	*
		24	−3.7	±0.3	−2.5	±0.5
		48	−3.9	±0.2	−7.1	±0.8
		72	−4.7	±0.4	*	*
5	Superoxide dismutase [*Raphanus sativus*]	48	*	*	−2.3	±0.3
		72	*	*	−1.9	±0.2
6	Glutathione-S-transferase [*Brassica rapa* subsp. pekinen*sis*]	48	+2.5	±0.6	*	*
		72	+2.6	±0.2	*	*
7	Chloroplast stem-loop binding protein-41 [*Arabidopsis* thaliana]	72	+1.8	±0.2	*	*
8	Chloroplast stem-loop binding protein-41 [*Arabidopsis* thaliana]	12	+1.9	±0.1	*	*
		72	+2.0	±0.2	−1.6	±0.0
9	Cysteine synthase [*Populus trichocarpa*]	12	+1.9	±0.2	*	*
		48	+1.4	±0.1	*	*
		72	+5.3	±1.7	*	*
10	S-adenosylmethionine synthetase [*Brassica rapa* subsp. *pekinensis*]	72	+5.9	±1.1	*	*
11	Monodehydroascorbate reductase [*Brassica rapa* subsp. *pekinensis*]	72	+4.0	±0.5	*	*
12	Malate dehydrogenase [*Arabidopsis thaliana*]	12	+2.4	±0.3	*	*
		72	+2.4	±0.5	*	*
13	Major latex-related protein [*Capsella rubella*]	12	+8.5	±3.8	*	*
		24	+2.1	±0.1	*	*
		48	+2.2	±0.3	−2.0	±0.0
		72	+1.3	±0.0	*	*
14	20 kDa chaperonin, chloroplastic [*Arabidopsis thaliana*]	12	+3.9	±0.4	*	*
		48	+3.6	±0.8	*	*
		72	+2.4	±0.8	*	*
15	Putative elongation factor P (EF-P) [*Arabidopsis thaliana*]	12	+3.0	±0.6	*	*
		24	−2.3	±0.0	*	*
		48	−3.6	±0.7	−3.0	±0.6
16	Putative uncharacterized protein At3g52150 [*Arabidopsis thaliana*]	48	*	*	−4.8	±0.1
17	ATP synthase (Fragment) [*Brassica campestris*]	12	+4.6	±1.6	*	*
		48	−3.3	±0.6	−3.5	±0.3
		72	−8.0	±0.0	*	*
18	RuBisCO large subunit-binding protein subunit alpha, chloroplastic [*Brassica napus*]	12	*	*	*	*
		24	*	*	−1.7	±0.2
		48	*	*	−2.1	±0.4
19	Cytochrome b6-f complex iron-sulfur subunit, chloroplastic [*Arabidopsis thaliana*]	24	+1.5	±0.1	*	*
20	Predicted protein Tax_Id = 3694 [*Populus trichocarpa*]	12	+2.0	±0.0	*	*
		24	+1.8	±0.1	*	*
		72	*	*	+2.5	±0.4
21	Eukaryotic translation initiation factor-5A [*Brassica napus*]	12	*	*	+3.9	±0.8
		24	−7.2	±1.3	*	*
22	Ribulose bisphosphate carboxylase large chain [Brassica juncea]	12	+5.5	±0.4	+3.1	±1.2
		48	+1.6	±0.1	*	*
23	Putative p-nitrophenylphosphatase [*Arabidopsis thaliana*]	12	+2.1	±0.1	*	*
		24	−1.6	±0.0	*	*
		48	*	*	−2.6	±0.3
		72	*	*	−1.6	±0.2
24	Chloroplast fructose-1,6-bisphosphatase I [*Fragaria ananassa*]	12	+2.6	±0.2	*	*
25	ATP synthase subunit beta [*Physalis* sp. P078]	12	+2.3	±0.1	+2.7	±0.2
26	Ribulose bisphosphate carboxylase large chain [*Leucas capensis*]	12	+5.0	±0.5	+4.1	±0.4
		48	+2.5	±0.2	*	*
27	Dihydrolipoyl dehydrogenase 1, mitochondrial [*Arabidopsis thaliana*]	12	+8.5	±0.9	+3.0	±0.3
28	Carbonic anhydrase, chloroplast [*Arabidopsis thaliana*]	12	+2.9	±0.2	*	*
		48	*	*	−2.2	±0.1
29	Putative uncharacterized protein [*Sclerotinia sclerotiorum*]	72	*	*	+0.03	±0.00
30	Elongation factor 1-beta [*Sclerotinia sclerotiorum*]	48	+0.01	±0.00	+0.01	±0.00
		72	+0.01	±0.00	+0.05	±0.01
31	Aspartate protease [*Sclerotinia sclerotiorum*]	48	*	*	+0.02	±0.00
		72	+0.01	±0.00	+0.09	±0.01
32	Putative uncharacterized protein [*Sclerotinia sclerotiorum*]	48	+0.01	±0.00	+0.03	±0.00
		72	+0.01	±0.00	+0.14	±0.01
33	Putative uncharacterized protein [*Sclerotinia sclerotiorum*]	48	*	*	+0.03	±0.00
		72	+0.01	±0.00	+0.25	±0.02
34	ATP synthase subunit beta [*Sclerotinia sclerotiorum*]	48	*	*	+0.05	±0.01
		72	*	*	+0.33	±0.01
35	Malate dehydrogenase [*Sclerotinia sclerotiorum*]	72	*	*	+0.21	±0.04
36	Glyceraldehyde 3-phosphate dehydrogenase [*Botryotinia fuckeliana*]	48	+0.07	±0.00	+0.06	±0.00
		72	+0.06	±0.00	+0.28	±0.04
37	Putative uncharacterized protein [*Sclerotinia sclerotiorum*]	72	0.04	±0.01	0.17	±0.02

A Spot numbers as given on the 2-D gel image (Figure 3) that were significantly affected in response to the pathogen challenge.

B hpi  =  hours post inoculation.

C Expression ratios (fold changes) for each protein was calculated from the average of spot intensities values of treatment with respect to their control genotype at each time point. However, spots 30–37 represent the actual values of the spot density data in the absence of detection of any protein in the control genotype (for these spots). These spot densities were measured from the filtered 2-DE images and each spot density value comprises the sum of the signal intensities (expressed as spot/optical density units) of all the pixels that make up the object.

D SE represents the standard error associated with the mean value of expression ratio for each spot and for each genotype at different time points separately.

A total of 28 proteins identified from the resistant Charlton and/or the susceptible RQ001-02M2 were classified into seven different functional categories. The protein functions were assigned based on the available literature and protein function database Pfam (pfam.sanger.ac.uk/) or InterPro (www.ebi.ac.uk/interpro/). A large proportion of the proteins (39%) identified that were modulated in response to the pathogen challenge were those involved in metabolism (including carbon and phosphorous metabolism), whereas 14% of the proteins could not be classified as their function was not known. The next largest group comprised enzymes involved in protein synthesis (14%), followed by a group having a role as antioxidants (11%) and those involved in protein folding and post-translation modification (11%). The remainder comprised pathogenesis-related proteins and proteins involved in ethylene biosynthesis and signaling (4% each) (Table 2).

#### Proteins of pathogen origin that were extracted from the infected tissue of the resistant Charlton and the susceptible RQ001-02M2

This study revealed nine enzymes which were of fungal origin and were most likely extracted from the infected tissues of the *B. napus* genotypes (Table 2; Table S1). Of these, the function of the four protein spots remains to be determined, whilst the majority of the remainder corresponded to enzymes involved in metabolic pathways. The amount of fungal proteins found corresponded directly with the expansion of lesion size and amount of fungus on the susceptible and resistant genotypes. For instance, higher abundance of aspartic protease was identified in the susceptible RQ001-02M2 with spot intensities of 0.02 and 0.09 at 48 and 72 hpi, respectively, in contrast to the spot intensity value of 0.01 at 72 hpi in the resistant Charlton. The maximum levels of all the fungal proteins were observed at 72 hpi in susceptible RQ001-02M2, when its cotyledons were fully covered by mycelial growth.

## Discussion

In this study, we conducted proteomics-based analysis involving 2-DE and histological investigations to get a better understanding of the defence responses in cotyledon tissues of resistant and susceptible genotypes of *B. napus* against *S. sclerotiorum*. Proteomics analysis is a valuable approach in unraveling molecular mechanisms against various stresses, as it provides continuity between genome sequence information with the protein profile that can indicates possible biochemical cellular pathways involved [29]. The necessity of such investigations is particularly evident for *S. sclerotiorum* with a host range encompassing over 400 plant species, in order for durable resistance to be designed. The current investigation identified the proteins that were differentially expressed between resistant Charlton and susceptible RQ001-02M2 when infected with *S. sclerotiorum*, such as those related to primary and secondary metabolic pathways (e.g. cytochrome b6-f complex, carbonic anhydrase, malate dehydrogenase), antioxidant defence (glutathione S- transferase, monodehydroascorbate reductase, superoxide dismutase), protein synthesis (e.g. cysteine synthase), pathogenesis related proteins (major latex-related protein), ethylene biosynthesis (S-adenosylmethionine synthase) and 20 kDa chaperonin. Additionally, a eukaryotic translation initiation factor 5A protein was identified, whose increased levels were found in susceptible RQ001-02M2 but with a decreased abundance in resistant Charlton. Below we discuss the potential role(s) of these proteins in mediating resistance against *S. sclerotiorum*. Matching changes in abundance of proteins associated with the changes at histological and disease progression levels offers a more comprehensive understanding of resistance mechanisms involved.

A number of chloroplastic proteins involved in plant primary metabolism showed increased abundance only in the proteome of the resistant Charlton in response to the pathogen challenge. These include cytochrome b6-f complex (Spot 19, 1.5-fold at 24hpi; Table 2), chloroplast stem-loop binding protein-41 (CSP41A; Spot 7, 1.8-fold at 72 hpi; Spot 8, 1.9-fold at 12 hpi and 2-fold at 72hpi), chloroplast fructose 1–6, biphosphastase (Spot 24, 2.6-fold at 12 hpi) and carbonic anhydrase (CA; Spot 28, 2.9-fold at 12hpi). Similarly, the calvin cycle enzyme ribulose bisphosphate carboxylase (Rubisco) enzyme was found to be up-regulated in resistant Charlton to a higher levels (spots 22 and 26 , ∼5.5-fold at 12 hpi and 1.6 fold at 48 hpi) as compared to susceptible RQ001-02M2 (∼3.1 fold at 12 hpi). Similar observations were found by Sharma et al. [39] where abundance of proteins involved in primary metabolism was enhanced in a *B. napus* line that was tolerant to necrotrophic pathogen *Alternaria brassicase*. Increased abundance of photosynthetic enzymes predominantly in resistant Charlton in our study suggests that induction of defence responses against *S. sclerotiorum* could be a cost-intensive process requiring an accelerated metabolic rate and an increased demand for assimilates as reported in other studies [40]. Additionally, CA, whose increased levels was evident only in the resistant Charlton, is reported to play an important role in defence responses, such as for a CA-silenced genotype of *Nicotiana benthamiana* was more susceptible to infection caused by *Phytophthora infestans*. Slaymaker et al. [41] also found that silencing of the CA gene in leaf tissue of *N. benthamiana* suppressed *Pto*:*avrPto-*mediated hypersensitive responses (HR). This is in agreement with our morphological and histological investigations as resistance reaction was only observed in the resistant Charlton, further supporting the potential involvement of CA in mediating resistance against *S. sclerotiorum*.

Another enzyme involved in the plant metabolic pathway is malate dehydrogenase (MDH), the levels of which were increased ∼2.5 fold only in the resistant Charlton in response to the pathogen challenge at 12 hpi. MDH is an enzyme of the tricarboxylic acid cycle and catalyses the conversion of malate into oxaloacetate, producing sufficient quantity of NAD(P)H, that can then be used to form H_2_O_2_ (responsible for oxidative stress), possibly by NAD(P)H oxidase on plasmalemma [42], [43]. Several past studies have demonstrated the increase in abundance of malate dehydrogenase in response to biotic and abiotic stresses [44], [45]. Similarly, increased abundance of mitochondrial dihydrolipoyl dehydrogenase (DLD) was observed in resistant Charlton at 12 hpi in our study at a much higher level (Spot 27, 8.5×) as compared with susceptible RQ001-02M2 (3.0x). DLD is a shared subunit of α-ketoglutarate and the pyruvate dehydrogenases complex, which catalyses NADH oxidation by oxygen with the concomitant formation of H_2_O_2_ [46], [47]. Tahara et al. [48] also demonstrated the role of DLD as a source of reactive oxygen species (ROS) in *Saccharomyces cerevisiae*, as the strains lacking the *LPD1* (lipoyl-dehydrogenase) gene prevented induction of oxidative stress. These results suggest that increase in the levels of MDH and DLD in our study could have led to the oxidative stress in resistant Charlton especially at an early stage of infection process as both genes were up-regulated at 12 hpi. Our results corroborate with the previous study of microarray analysis of the *B. napus*-*S. sclerotiorum* pathosystem by Zhao et al. [22], who suggested that the changes in expression of genes encoding enzyme involved in carbohydrate and energy metabolism are directed towards shuttling carbon reserve to TCA cycle, which as a consequence generates ROS. Oxidative stress leads to the production of ROS, a known key event relating to HR in restricting hyphal growth and reinforcing the cell; and it also acts as a diffusible signal for induction of cellular protectant genes [49], [50]. It is interesting that similar responses, such as impeded fungal growth and up-regulation of cellular protectant genes, have been found only in resistant Charlton, further indicating the potential roles of MDH and DLD in mediating defence responses response against *S. sclerotiorum*.

Although, ROS generated in response to various biotic and abiotic stresses is known as a key event relating to HR, it is, however, needed to be processed rapidly because of their ability to cause oxidative damage to proteins, DNA and lipids [51]. In order to keep ROS below threshold levels compatible for cell metabolism, plants possess a battery of both enzymatic and a non-enzymatic ROS-detoxifying mechanisms [52]. In our study, the resistant Charlton exhibited significant increases in the levels of both glutathione S-transferase (GST) (Spot 6, 2.5-fold at 48 hpi; 2.6-fold at 72 hpi; Table 2) and cysteine synthase (Spot 9, 1.9-fold at 12 hpi; 1.4-fold at 48 hpi; 5.3-fold at 72hpi) in response to the pathogen challenge. Interestingly, these proteins could not be detected in susceptible RQ001-02M2 or its mock-inoculated control samples. GSTs play a role as cellular protectants and prevent oxidative damage, and are known to be induced in response to the various biotic and abiotic stresses including against *S. sclerotiorum* [18], [22], [53]. Cysteine synthase on the other hand is a key enzyme that catalyses cysteine biosynthesis and is incorporated into different kinds of proteins and/or acts as a precursor for a range of sulfur-containing metabolites [54], [55]. Importantly, cysteine is involved in the biosynthesis of tripeptide glutathione (GSH), which is an important universal antioxidant (or detoxifier of ROS) [56]. Over-expression of cysteine synthase has been reported to increase both cysteine and GSH in *N. tabacum* [54]. Similarly, Mauch and Dudler [57] found enhanced GSH activity with increased levels of glutathione S-transferases (GSTs). Increased abundance of cysteine synthase together with GSTs suggests an increase in GSH content and hence antioxidant defence prevailing only in the resistant Charlton.

In addition to antioxidant defence, elevated levels of GSTs and cysteine synthase are also known to stimulate transcription of other defence genes including those that encode: (a) the phenylpropanoid biosynthetic enzymes phenylalanine ammonia lyase (PAL) and chalcone synthase (CHS) that are involved in lignin (PAL) and phytoalexin (PAL, CHS) production; (b) cell wall hydroxyproline-rich glycoproteins; [58], [59]; and, (c) various pathogenesis-related proteins such as chitinase or β-glucanse [59]–[61]. The anatomical investigations of the resistant Charlton in this study further confirms up-regulation of these enzymes as a number of darkly-stained areas around the dead cells of palisade mesophyll layer were evident, indicating accumulation of polyphenolic and/or phytoalexin compounds (Figure 2E). Similar observations were also made by Hua Li et al. [36] in association with resistance of *B. napus* to *Leptosphaeria maculans* and, by Eynck et al. [24] where induced lignification in the immediate vicinity of the infection site of the stems of *Camelina sativa* was reported during histochemical analysis as a resistance response to *S. sclerotiorum.* Similarly, impeded fungal growth and increased hyphal cell diameter (and hyphal swellings) were observed on the cotyledons of the resistant Charlton at 48 and 72 hpi, but not in the susceptible RQ001-02M2. Hyphal swellings and vacuolation of the mycelial content have also been observed in the interaction of *S. sclerotiorum* with *Pseudomonas cepacia*, and antifungal compounds released by *P. cepacia* were found to be responsible for such abnormalities [62]. It is possible that the enhanced level of GSTs and cysteine synthase may have induced the production and release of various hydrolytic enzymes and/or antifungal proteins, leading to the observed impeded fungal growth and hyphal swellings on the cotyledons of the resistant Charlton. Previous studies have also indicated that the change in the expression/activity of ROS-scavenging enzymes could be a key step in the activation of defence mechanism(s) against various phytopathogens [52], [56], [63].

There was significant increase in the levels of another antioxidant enzyme, monodehydroascorbate reductase (MDHAR) that was found only in resistant Charlton at 72 hpi (∼4.0-fold) in response to the pathogen challenge. MDHAR is an important component of the ascorbate-glutathione cycle and can be directly reduced to ascorbate within the cell such at the plasmalemma or at thylakoid membrane [56]. Ascorbate is considered as the most important reducing substrate for H_2_O_2_ detoxification [56]. MDHAR is also capable of reducing phenoxyl radicals to their respective parental phenols that are potent antioxidants with an activity equivalent to ascorbate in relation to detoxification of ROS [64]. Increased activity of MDHAR only in the resistant Charlton, indicates that the antioxidant defence can mediate resistance responses against *S. sclerotiorum*.

Whilst the susceptible RQ001-02M2 exhibited significant ∼2-fold decrease in the levels of superoxide dismutase (SOD), at 48 and 72 hpi, no significant changes in the abundance of this enzyme were found in the resistant Charlton. SOD is one of the main components of the ROS scavenging machinery of the plant defence system [65]. The significantly lower levels of SOD in susceptible RQ001-02M2 at 48 and 72 hpi, indicate a decrease in the levels of antioxidant defence in response to the pathogen challenge in this genotype, and hence enhanced oxidative damage and/or localized cell death. This is corroborated by the morphological studies of the susceptible RQ001-02M2, where increase in the expansion of cotyledon lesion diameter at 48 hpi and 72 hpi correlated with the decrease in the levels of SOD. Our anatomical investigations revealed cytoplasmic disorganization of the palisade mesophyll cells in the susceptible genotype underneath an intact epidermis, indicating cell death in response to pathogen invasion. Thus, enhanced cell death due to decreased ROS metabolism in response to the pathogen invasion may have aided the infection of and colonization by *S. sclerotiorum* by providing nutrients needed by the pathogen. Previous studies in *A. thaliana* have also established that it is the increased levels of accumulated (or generated) superoxide in response to the pathogen challenge that facilitates infection caused by nectrotrophic pathogens such as *S. sclerotiorum* [66], [67]. Together, these findings suggest that the ROS scavenging mechanism(s) of the susceptible RQ001-02M2 would have been countered during pathogen invasion by one or more toxic metabolites produced by *S. sclerotiorum*, in a way similar to the observation by Liang et al. [68], who found suppressed SOD activity in *B. napus* from exogenously supplied oxalic acid.

Another protein, eukaryotic translation initiation factor 5A (elF 5A), was found to be up-regulated only in susceptible RQ001-02M2 by 3.9-fold at 12 hpi as compared to resistant Charlton where its decrease abundance was observed by 7.2-fold at 24 hpi. The elF 5A is a highly conserved protein found in all eukaryotic organisms and various investigations at biochemical and molecular levels have revealed that it is the only protein containing the post-translationally synthesized amino acid hypusine [69], [70]. This enzyme is considered to be a fundamental requirement for plant growth and development by regulating cell division, cell growth, and cell death as shown in *A. thaliana* by Feng et al. [69]. Similarly, Hopkins et al. [70] reported that AtelF5A-2, one of the three elF5A genes in *A. thaliana* regulate program cell death caused by infection with the nectrotrophic pathogen *P. syringae*. They found that transgenic *A. thaliana* plants with constitutively suppressed AteIF5A-2 exhibited marked resistance to programmed cell death induced by virulent *Pseudomonas syringae*, and there was a corresponding reduction in pathogen growth and development of disease symptoms in the plant tissue. These results corroborate with our study where resistant Charlton with decreased abundance in elF 5A exhibited significantly reduced disease symptoms and pathogen development both at morphological and histological levels (Figure 1 and Figure 2). However, increase in abundance of elF 5A in susceptible RQ001-02M2, especially at an early stage of infection process, may have led to the cell death observed, further facilitating the establishment and development of *S. sclerotiorum*, especially as it is a necrotrophic pathogen.

Pathogenesis-related (PR) proteins comprise one of the important components of the inducible repertoire of the plant self-defence mechanisms that are produced in response to the invading pathogen and/or abiotic stresses [71]. The major latex-related proteins (MLP) detected in our study were assigned to the PR-10 family on the basis of their sequence homology [71], [72]. PR-10 proteins are involved in defence responses because of their ribonucleolytic, antifungal and antibacterial activities [73]. We found that the MLPs were up-regulated (8.5-fold at 12hpi, 2.1-fold at 24 hpi and 2.2-fold at 48 hpi) in the resistant Charlton but it was down-regulated by 2-fold in susceptible RQ001 at 48 hpi, indicating its potential role in mediating defence responses against *S. sclerotiorum*. Calla et al. [19] have also reported homologs of genes encoding PR-10 in soybean stem tissue in response to the *S. sclerotiorum* infection. Further, impeded fungal growth and disintegration of hyphal cell walls observed only on resistant Charlton in our study in histological examinations, could also have been associated with the antifungal property of MLPs.

Increased abundance of S-adenosylmethionine synthetase (AdoMet synthetase) was found in the resistant Charlton (by 5.9-fold at 72 hpi), in contrast to the susceptible RQ001-02M2 in which no significant increase in the levels of this enzyme was observed following inoculation. AdoMet synthetase catalyses the biosynthesis of S-adenosylmethionine (AdoMet), a precursor molecule of ethylene (ET) [74], [75] and polyamines [76]. ET plays an important role in the activation of various defence responses, such as induction of PR proteins and the synthesis of phytoalexin against various microbial pathogens [77]. A previous report by Liang et al. [25] on the compatible interaction in the *B. napus*–*S. sclerotiorum* pathosystem found decreased levels of methionine adenosyltransferase, which is responsible for the catalysis of AdoMet, and suggested a possible role of ET in mediating responses of *Brassica* spp. to the challenge by *S. sclerotiorum*. Yang et al. [23] also found that transgenic canola producing low levels of ET was relatively more susceptible to *S. sclerotiorum* as compared with its wild-type counterpart. Interestingly, cysteine, which was found in higher levels only in the resistant Charlton also acts as a sulfur donor of the amino-acid methionine, an immediate precursor of S-adenosylmethionine [78]. The increased levels of AdoMet synthetase only in resistant Charlton in this study supports previous reports that ET signaling plays an important role in mediating defence responses of *B. napus* against *S. sclerotiorum.*

Chaperones assist with protein refolding under stress conditions and their levels are known to be affected in many studies investigating both compatible and incompatible plant-pathogen interactions [79], [80]. The present study revealed three proteins with chaperone activity, *viz.* 20 kDa chaperonin, protein disulfide isomerase (PDI) and protein grpE (spots 2, 3 and 14), that were modulated in response to the pathogen challenge. The abundance of a 20 kDa chaperonin was significantly increased only in the resistant Charlton at 12 hpi, 48 hpi and 72 hpi in response to the pathogen challenge. Interestingly, Liang et al. [25] observed a decrease in the levels of the enzyme chaperonin in compatible interactions in *B. napus*-*S. sclerotiorum* pathosystem, which further suggests that chaperonin mediates defence responses against this pathogen. Similarly, levels of grpE, which is a co-chaperone of heat-shock proteins (Hsps), decreased in response to the pathogen challenge in the susceptible RQ001-02M2 at 48 hpi. However, the levels of PDI in this study were significantly increased in both resistant and susceptible genotypes at 72 hpi, in contrast to the observation of Liang et al. [25] of decreased levels of PDI in a susceptible *B. napus* genotype in response to *S. sclerotiorum* infection. Increased PDI in both the resistant and the susceptible genotypes in our study suggests that the activity of PDI was probably modulated in response to the stress conditions, rather than mediating defence responses in response to the *S. sclerotiorum* infection.

## Conclusions

The present study utilizes the cotyledon assay to identify the differentially expressed proteins in resistant Charlton and susceptible RQ001-02M2 against *S. sclerotiorum*. Interestingly, our results of change in protein expression profile in cotyledon tissue of *B. napus* are similar with the previous microarray studies conducted at leaf/seedling stage [18], [21] or at stem stage [22] of *B. napus* against *S. sclerotiorum*, and in other host species of this pathogen such as soybean [19]. The similarity of our results with previous microarray studies, especially with those conducted in infected stems [22] which is a natural site for infection in this pathosystem, validates further the potential of cotyledon assay for screening purposes, particularly within *B. napus*. Differences observed in resistance responses between cotyledon and stem tissue of *B. napus* identified in our previous study [32], could be largely attributed to the environmental factors, known to have a major impact on the manifestation of this disease. Our proteomic investigation demonstrates a coordinated increased in expression of proteins only in resistant Charlton, such as those related to primary and secondary metabolic pathways (e.g. CA, MDH), antioxidant defence (e.g. GST, SOD), protein synthesis (cysteine synthase), ethylene biosynthesis (AdoMet synthetase) and pathogenesis related proteins (MLPs), have a significant role in mediating the defence responses against *S. sclerotiorum*. In the susceptible genotype, decreased levels of these enzymes, especially those involved in antioxidant defence, and increased abundance of elF 5A and DLD (at an early stage of infection process) in response to the pathogen infection, together have some role in manifesting the cell death observed in this genotype. Our anatomical investigations also revealed extensive cytoplasmic disorganization of the palisade mesophyll cells in the susceptible genotype as compared to resistant Charlton underneath an intact epidermis, indicating cell death in response to pathogen invasion. The cell death caused by these proteins in the susceptible RQ001-02M2 was in fact advantageous to this necrotrophic pathogen as it assists in sustaining pathogen growth through provision of adequate nutrients. However, increased levels of the enzymes such as MDH and DLD, that could be responsible for ROS generation in the resistant Charlton, were mainly manifested at an early stage of the infection process. Any selective advantage of localized cell death to the pathogen in the resistant Charlton was prevented by increased levels of other defence-related enzymes, such as those involved in ROS detoxification (MDHAR), cellular protectants (GST) and cysteine synthase (which was up-regulated at an early stage of infection), and decreased levels of elF 5A enzyme. These enzymes likely stimulated the transcription of various phytoalexins and pathogenesis-related proteins (such as MLPs in our study) that prevented the spread of the pathogen within the host tissue. These findings were supported by our anatomical studies in which darkly-stained areas surrounding the dead cells in the palisade mesophyll layer (indicative of phytoalexins and/or phenolics compounds) were only observed in the resistant Charlton. Engineering *B. napus* plants to over-express the enzymes that were only up-regulated in the resistant Charlton would be a novel and effective strategy for enhancing resistance against this pathogen.

## Supporting Information

Figure S1 **Mean values of cotyledon lesion diameter for resistant and susceptible** ***Brassica napus*** **genotypes, over time.** Spring type *B. napus* resistant Charlton and susceptible RQ001-02M2 were inoculated with *Sclerotinia sclerotiorum* isolate MBRS-5. Mean values of cotyledon lesion diameter (mm) were measured at 24, 48, 72 and 96 days post inoculation (dpi). Bar on each value represents standard error associated with mean value of cotyledon lesion diameter.
(TIF)

Figure S2 **Closer views of the gels images showing significant changes.** Differentially expressed proteins were identified in *Brassica napus* resistant Charlton and susceptible RQ001-02M2 through two dimension gel electrophoresis. The numbers shown correspond with the spot numbers mentioned in Table 2 and in Table S1. Where R, Rc, S and Sc represent resistant (cv. Charlton), resistant control (mock inoculated resistant cultivar), susceptible (cv. RQ001-02M2) and susceptible control (cv. mock inoculated susceptible cultivar), respectively. Spot images are taken from the representative gels of 72 hours post inoculation (hpi) for 1–13 proteins; 48 hpi for 14–17; 24hpi for 18–21 and at 12 hpi for 22–28 proteins.
(TIF)

Figure S3 **Closer views of protein spots of pathogen origin.** Nine protein spots extracted from the infected tissue of *Brassica napus* genotypes were identified to be of pathogen origin (*Sclerotiorum sclerotiorum)*. The numbers shown correspond with the spot numbers given in Table 2. Where R, and S represent resistant (cv. Charlton) and susceptible (cv. RQ001-02M2) genotypes, respectively. Spot images for these proteins are taken from the representative gels at 72 hours post inoculation.
(TIF)

Table S1 **Details of the Mascot analysis.** Differentially expressed proteins of *Brassica napus* resistant Charlton and susceptible RQ001-02M2 at various times after inoculation with *Sclerotinia sclerotiorum* were analysed by MALDI TOF/TOF (electrospray ionisation MS/MS) mass spectrometer. Mass spectra were analysed to identify protein(s) of interest using Mascot sequence matching software with Ludwig NR Database. The protein spots were identified as being ‘significant hit’ (P<0.05) based on individual peptide ion score.
(DOCX)
