# Supplementary figures and images for: Differentially Expressed Proteins and Associated Histological and Disease Progression Changes in Cotyledon Tissue of a Resistant and Susceptible Genotype of Brassica napus Infected with Sclerotinia sclerotiorum
Source: PLoS One. 2013 Jun 11;8(6):e65205. doi: 10.1371/journal.pone.0065205 (PMC3679123; doi:10.1371/journal.pone.0065205)

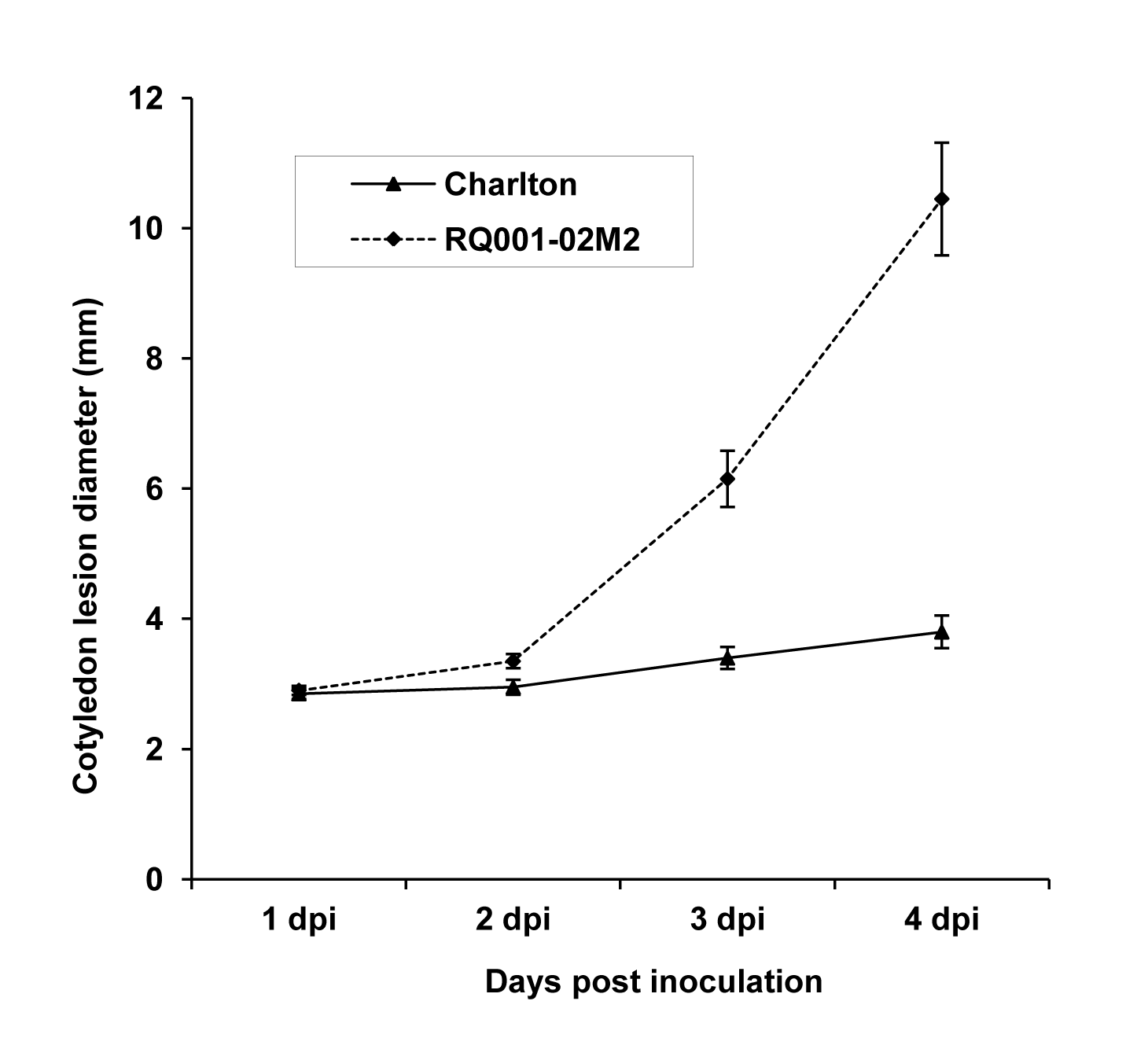

Supplement: Figure S1 — Mean values of cotyledon lesion diameter for resistant and susceptible Brassica napus genotypes, over time. Spring type B. napus resistant Charlton and susceptible RQ001-02M2 were inoculated with Sclerotinia sclerotiorum isolate MBRS-5. Mean values of cotyledon lesion diameter (mm) were measured at 24, 48, 72 and 96 days post inoculation (dpi). Bar on each value represents standard error associated with mean value of cotyledon lesion diameter. (TIF) [file pone.0065205.s001.tif]

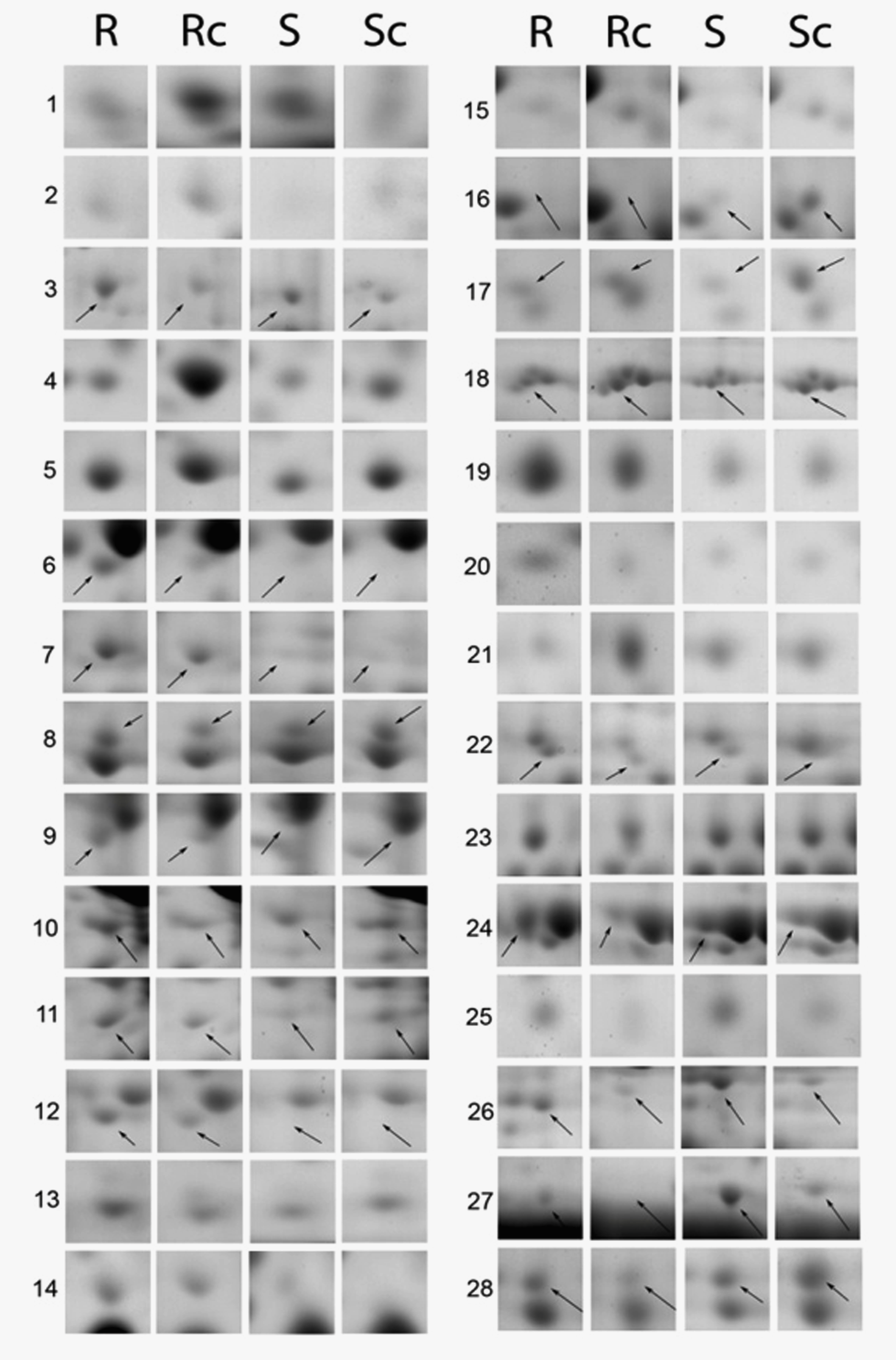

Supplement: Figure S2 — Closer views of the gels images showing significant changes. Differentially expressed proteins were identified in Brassica napus resistant Charlton and susceptible RQ001-02M2 through two dimension gel electrophoresis. The numbers shown correspond with the spot numbers mentioned in Table 2 and in Table S1. Where R, Rc, S and Sc represent resistant (cv. Charlton), resistant control (mock inoculated resistant cultivar), susceptible (cv. RQ001-02M2) and susceptible control (cv. mock inoculated susceptible cultivar), respectively. Spot images are taken from the representative gels of 72 hours post inoculation (hpi) for 1–13 proteins; 48 hpi for 14–17; 24hpi for 18–21 and at 12 hpi for 22–28 proteins. (TIF) [file pone.0065205.s002.tif]

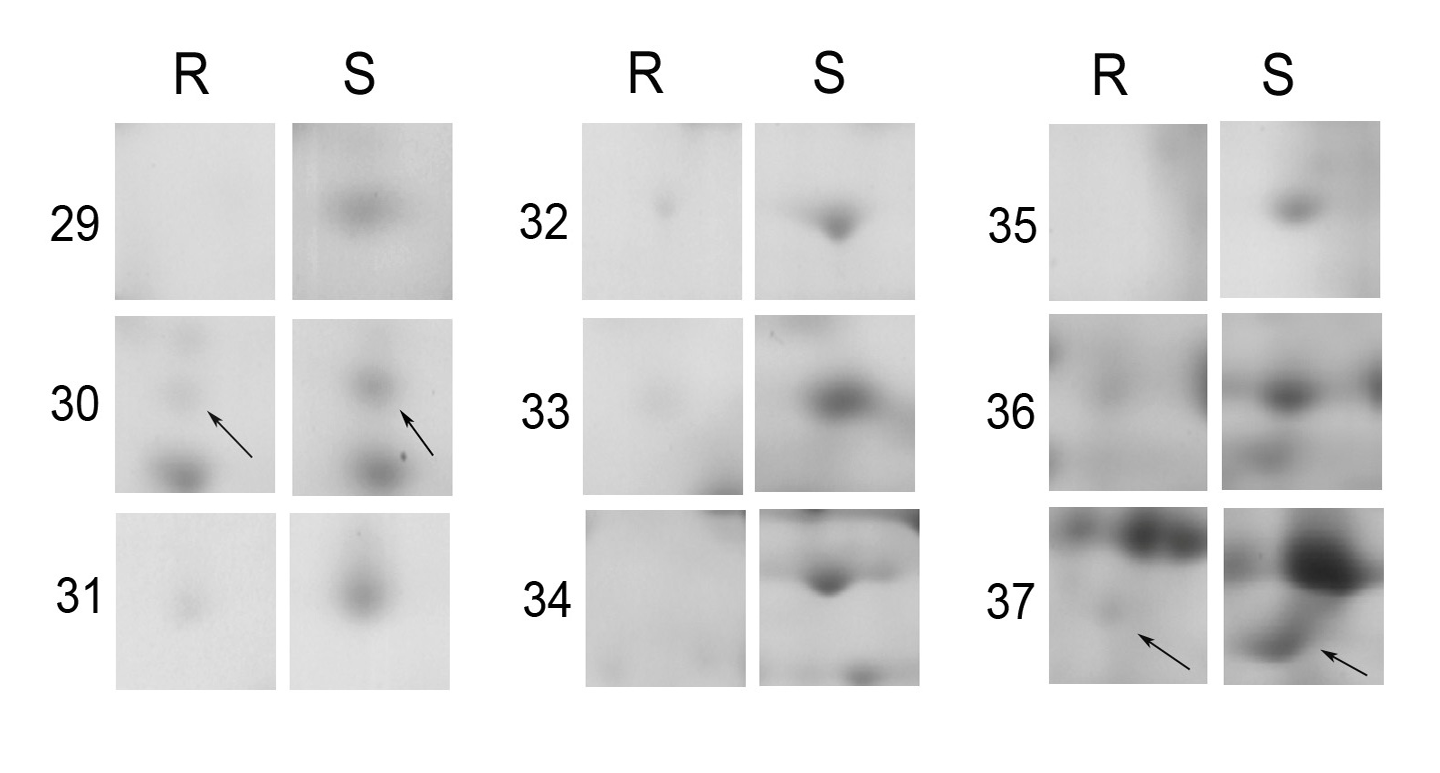

Supplement: Figure S3 — Closer views of protein spots of pathogen origin. Nine protein spots extracted from the infected tissue of Brassica napus genotypes were identified to be of pathogen origin (Sclerotiorum sclerotiorum). The numbers shown correspond with the spot numbers given in Table 2. Where R, and S represent resistant (cv. Charlton) and susceptible (cv. RQ001-02M2) genotypes, respectively. Spot images for these proteins are taken from the representative gels at 72 hours post inoculation. (TIF) [file pone.0065205.s003.tif]
