# Supplementary material for: Differentially Expressed Proteins and Associated Histological and Disease Progression Changes in Cotyledon Tissue of a Resistant and Susceptible Genotype of Brassica napus Infected with Sclerotinia sclerotiorum
Source: PLoS One. 2013 Jun 11;8(6):e65205. doi: 10.1371/journal.pone.0065205 (PMC3679123; doi:10.1371/journal.pone.0065205)
Supplement: Table S1 — Details of the Mascot analysis. Differentially expressed proteins of Brassica napus resistant Charlton and susceptible RQ001-02M2 at various times after inoculation with Sclerotinia sclerotiorum were analysed by MALDI TOF/TOF (electrospray ionisation MS/MS) mass spectrometer. Mass spectra were analysed to identify protein(s) of interest using Mascot sequence matching software with Ludwig NR Database. The protein spots were identified as being ‘significant hit’ (P<0.05) based on individual peptide ion score. (DOCX) [file pone.0065205.s004.docx]

**Table S1**: **Details of the Mascot analysis.** Differentially expressed proteins of *Brassica napus* resistant Charlton and susceptible RQ001-02M2 at various times after inoculation with *Sclerotinia sclerotiorum* were analysed by MALDI TOF/TOF (electrospray ionisation MS/MS) mass spectrometer. Mass spectra were analysed to identify protein(s) of interest using Mascot sequence matching software with Ludwig NR Database. The protein spots were identified as being ‘significant hit’ (P <0.05) based on individual peptide ion score.

| **Spot No. ^A^** | **Protein name** | **% SC (NP) ^B^** | **Score^C^** | **Peptide Sequence^D^** | **Accession no. ^E^** | **Exp MW/pI (kDa/pI) ^F^** | **Theo**  **MW/pI (kDa/pI) ^G^** |
| --- | --- | --- | --- | --- | --- | --- | --- |
| 1 | 50S ribosomal protein L12-C [Arabidopsis thaliana] | 12 (2) | 201/54 | K.IGSEISSLTLEEAR.I | Q8LBJ7 | 16.6/4.9 | 19.7/6.0 |
|  |  |  |  | R.ILVDYLQDK.F |  |  |  |
| 2 | Protein grpE [Prochlorococcus marinus] | 3 (1) | 70/53 | R.ISADFDNFR.K | A2BNE2 | 35.7/4.6 | 27.5/4.6 |
| 3 | Protein disulfide isomerase [Brassica carinata] | 37 (14) | 681/53 | K.IQGFPTIK.I | Q38HW3 | 56/5.1 | 55.9/5.0 |
|  |  |  |  | K.VVVYEGSR.T |  |  |  |
|  |  |  |  | K.GFPTIYFR.S |  |  |  |
|  |  |  |  | K.SIQDYNGPR.E |  |  |  |
|  |  |  |  | R.TKEDFISFIEK.N |  |  |  |
|  |  |  |  | R.ADYDFAHTLDAK.L |  |  |  |
|  |  |  |  | R.LFKPFDELFVDSK.D |  |  |  |
|  |  |  |  | K.AAAELSSQSPPIFLAK.I |  |  |  |
|  |  |  |  | K.LSGEEFDSFMAVAEK.L |  |  |  |
|  |  |  |  | K.LDATANDIPSDTFDVK.G |  |  |  |
|  |  |  |  | K.IDASEESNKGIANEYK.I |  |  |  |
|  |  |  |  | K.NVLIEFYAPWCGHCQK.L |  |  |  |
|  |  |  |  | K.LAPILDEVALAFQNDPSVIVAK.L |  |  |  |
|  |  |  |  | K.ESSIPLVTVFDKDPSNHPYVSK.F |  |  |  |
| 4 | Light-harvesting complex I chlorophyll a/b binding protein 1 At3g54890.4 [Arabidopsis thaliana] | 12 (5) | 275/54 | K.ESELIHCR.W | A8MS75 | 21.1/5.7 | 23.4/6.9 |
|  |  |  |  | R.YKESELIHCR.W |  |  |  |
|  |  |  |  | K.YPGGAFDPLGYSK.D |  |  |  |
|  |  |  |  | K.KYPGGAFDPLGYSK.D |  |  |  |
|  |  |  |  | K.YPGGAFDPLGYSKDPK.K |  |  |  |
| 5 | Superoxide dismutase [Raphanus sativus] | 19 (5) | 242/53 | R.DFTSYEK.F | O65327 | 22.7/6.2 | 23.8/6.0 |
|  |  |  |  | R.AYVDNLKK.Q |  |  |  |
|  |  |  |  | K.QTLEFHWGK.H |  |  |  |
|  |  |  |  | K.QTLEFHWGKHHR.A |  |  |  |
|  |  |  |  | K.TFMNNLVSWEAVSSR.L |  |  |  |
| 6 | Glutathione-S-transferase [Brassica rapa subsp. pekinensis] | 42(10) | 525/54 | R.VLLTLHEK.N | Q5DNA8 | 24.5/6.1 | 24.3/5.7. |
|  |  |  |  | R.AITQYIAHR.Y |  |  |  |
|  |  |  |  | K.VPAFEDGDLK.L |  |  |  |
|  |  |  |  | R.RVLLTLHEK.N |  |  |  |
|  |  |  |  | K.VFGHAASTATR.R |  |  |  |
|  |  |  |  | K.LATVLDVYEAR.L |  |  |  |
|  |  |  |  | K.NLDFELVHVELK.D |  |  |  |
|  |  |  |  | R.NPFGKVPAFEDGDLK.L |  |  |  |
|  |  |  |  | K.VPAFEDGDLKLFESR.A |  |  |  |
|  |  |  |  | K.LFYGMTTDQAVVEEEEAK.L |  |  |  |
| 7 | Chloroplast stem-loop binding protein-41 chloroplastic CSP41A Tax_Id=3702 [Arabidopsis thaliana] | 22 (8) | 391/54 | K.DLLGWESK.T | Q9LYA9 | 40.6/6.2 | 44.1/8.5 |
|  |  |  |  | R.NMHFYAEPR.A |  |  |  |
|  |  |  |  | K.TVEIVHYDPK.A |  |  |  |
|  |  |  |  | K.TNLPEDLKER.F |  |  |  |
|  |  |  |  | K.DCEEWFFDR.I |  |  |  |
|  |  |  |  | K.QFLFISSAGIYK.S |  |  |  |
|  |  |  |  | K.DLDTVRPVVDWAK.S |  |  |  |
|  |  |  |  | K.NVLIVNTNSGGHAVIGFYFAK.E |  |  |  |
| 8 | Chloroplast stem-loop binding protein-41 chloroplastic CSP41A Tax_Id=3702 [Arabidopsis thaliana] | 15 (7) | 336/54 | R.FEEYVK.I | Q9LYA9 | 40.9/6.4 | 44.1/8.5 |
|  |  |  |  | K.ERFEEYVK.I |  |  |  |
|  |  |  |  | R.NMHFYAEPR.A |  |  |  |
|  |  |  |  | K.TVEIVHYDPK.A |  |  |  |
|  |  |  |  | K.DCEEWFFDR.I |  |  |  |
|  |  |  |  | K.QFLFISSAGIYK.S |  |  |  |
|  |  |  |  | K.DLDTVRPVVDWAK.S |  |  |  |
| 9 | Cysteine synthase [Populus trichocarpa] | 6 (2) | 143/53 | K.VTEGCGAYIAAK.Q | A9PGL6 | 38.8/6.9 | 40.4/8.6 |
|  |  |  |  | K.LIVTIHASFGER.Y |  |  |  |
| 10 | S-adenosylmethionine synthetase [Brassica rapa subsp. pekinensis] | 23 (7) | 468/43 | R.WLRPDGK.T | Q5DNB1 | 47.4/6 | 43.6/5.7 |
|  |  |  |  | K.TAAYGHFGR.D |  |  |  |
|  |  |  |  | K.TIFHLNPSGR.F |  |  |  |
|  |  |  |  | R.FVIGGPHGDAGLTGR.K |  |  |  |
|  |  |  |  | K.TNMVMVFGEITTK.A |  |  |  |
|  |  |  |  | R.SIGFISDDVGLDADKCK.V |  |  |  |
|  |  |  |  | R.VHTVLISTQHDETVTNDEIAR.D |  |  |  |
| 11 | Monodehydroascorbate reductase [Brassica rapa subsp. pekinensis] | 34 (10) | 591/43 | K.GYLFPEGAAR.L | Q93X74 | 45.9/6 | 46.6/5.8 |
|  |  |  |  | R.RVEHVDHSR.K |  |  |  |
|  |  |  |  | R.FGAYWVQDGK.V |  |  |  |
|  |  |  |  | K.EAVAPYERPALSK.G |  |  |  |
|  |  |  |  | K.YIILGGGVSAGYAAK.E |  |  |  |
|  |  |  |  | K.AVVVGGGYIGLELSAALR.I |  |  |  |
|  |  |  |  | K.EFASQGVKPGELAVISK.E |  |  |  |
|  |  |  |  | K.TSVPDVYAVGDVATFPLK.M |  |  |  |
|  |  |  |  | K.GTVASGFTAHPNGEVNEVQLK.D |  |  |  |
|  |  |  |  | K.AAEGGGAVEEYDYLPFFYSR.S |  |  |  |
| 12 | Malate dehydrogenase [Arabidopsis thaliana] | 12 (3) | 251/43 | R.DDLFNINAGIVK.N | A8MQK3 | 38.3/6.2 | 33.3/9.5 |
|  |  |  |  | K.KLFGVTTLDVVR.A |  |  |  |
|  |  |  |  | K.ALEGADLVIIPAGVPR.K |  |  |  |
| 13 | Major latex-related protein [Capsella rubella] | 23 (3) | 121/42 | R.GLEGHVMEQLK.V | B2WS86 | 16.7/6.1 | 17.7/5.7 |
|  |  |  |  | K.VYDVIYQFIPK.S |  |  |  |
|  |  |  |  | R.SWNYTWDGKEEMFK.E |  |  |  |
| 14 | 20 kDa chaperonin, chloroplastic [Arabidopsis thaliana] | 23 (5) | 350/43 | K.DLKPLNDR.V | O65282 | 24.1/5.6 | 26.8/8.9 |
|  |  |  |  | K.DGSNYIALR.A |  |  |  |
|  |  |  |  | K.YTSIKPLGDR.V |  |  |  |
|  |  |  |  | K.YAGTEVEFNDVK.H |  |  |  |
|  |  |  |  | K.EKPSIGTVIAVGPGSLDEEGK.I |  |  |  |
| 15 | Putative elongation factor P (EF-P) [Arabidopsis thaliana] | 18 (4) | 209/53 | K.VVDVDPGLR.G | Q8VZW6 | 23.2/5.4 | 26.4/8.6 |
|  |  |  |  | R.NYVNGSTVER.T |  |  |  |
|  |  |  |  | R.VLEFLHVKPGK.G |  |  |  |
|  |  |  |  | K.AGTNIEVDGAPWR.V |  |  |  |
| 16 | Putative uncharacterized protein At3g52150 [Arabidopsis thaliana] | 13 (5) | 121/43 | R.FGFATMK.S | Q8VYM4 | 28.7/5.6 | 27.7/8.9 |
|  |  |  |  | R.FGFATMK.S |  |  |  |
|  |  |  |  | R.VYIGNIPR.T |  |  |  |
|  |  |  |  | R.RVYIGNIPR.T |  |  |  |
|  |  |  |  | K.LVEEHGAVEKVQVMYDK.Y |  |  |  |
| 17 | ATP synthase (Fragment) [Brassica campestris] | 40 (4) | 258/43 | K.ITDTQLAEVR.S | Q39409 | 21.3/5.6 | 14.9/9.2 |
|  |  |  |  | K.LEPPQLAQIAK.Q |  |  |  |
|  |  |  |  | K.TVLDPSLVAGFTIR.Y |  |  |  |
|  |  |  |  | K.KQLEDIAAQLELGEIQLAA.- |  |  |  |
| 18 | RuBisCO large subunit-binding protein subunit alpha, chloroplastic [Brassica napus] | 19 (7) | 352/53 | K.LLVEFENAR.V | P21239 | 55.5/4.9 | 57.7/4.8 |
|  |  |  |  | R.NVVLDEFGSPK.V |  |  |  |
|  |  |  |  | K.ITAIKDIIPILEK.T |  |  |  |
|  |  |  |  | R.GYISPQFVTNPEK.L |  |  |  |
|  |  |  |  | R.AIELPDAMENAGAALIR.E |  |  |  |
|  |  |  |  | K.DSTTLIADAASKDELQAR.I |  |  |  |
|  |  |  |  | K.ALVAPAALIAQNAGIEGEVVVEK.I |  |  |  |
| 19 | Cytochrome b6-f complex iron-sulfur subunit, chloroplastic [Arabidopsis thaliana] | 17 (3) | 217/53 | K.GDPTYLVVENDK.T | Q9ZR03 | 18.0/6.2 | 24.6/8.8 |
|  |  |  |  | K.VLFVPWVETDFR.T |  |  |  |
|  |  |  |  | K.FLCPCHGSQYNAQGR.V |  |  |  |
| 20 | Predicted protein Tax_Id=3694 [Populus trichocarpa] | 5 (1) | 76/43 | R.FCDYTNDKSNLK.G | B9I1Y5 | 16.2/5.2 | 26.4/8.1 |
| 21 | Eukaryotic translation initiation factor-5A [Brassica napus] | 25 (3) | 154 /54 | K.TYPQQAGNIR.K | Q6RJS1 | 18.0/5.9 | 17.3/5.7 |
|  |  |  |  | K.CHFVAIDIFTAK.K |  |  |  |
|  |  |  |  | K.KLEDIVPSSHNCDVPHVNR.I |  |  |  |
| 22 | Ribulose bisphosphate carboxylase large chain [Brassica juncea] | 14 (7) | 296/53 | K.NHGMHFR.V | Q6Y9Y8 | 46.7/6.7 | 53.4/5.9 |
|  |  |  |  | K.DTDILAAFR.V |  |  |  |
|  |  |  |  | R.DNGLLLHIHR.A |  |  |  |
|  |  |  |  | R.ESTLGFVDLLR.D |  |  |  |
|  |  |  |  | R.FLFCAEAIYK.S |  |  |  |
|  |  |  |  | K.LNYYTPEYETK.D |  |  |  |
|  |  |  |  | K.TFQGPPHGIQVER.D |  |  |  |
| 23 | Putative p-nitrophenylphosphatase [Arabidopsis thaliana] | 3 (10) | 611/53 | R.YFNYYK.I | Q8GY27 | 33.7/5.0 | 34.3/5.1 |
|  |  |  |  | K.IQPDFYTSK.I |  |  |  |
|  |  |  |  | K.IQYGTLCIR.E |  |  |  |
|  |  |  |  | K.VYVIGEEGILK.E |  |  |  |
|  |  |  |  | K.RLVFVTNNSTK.S |  |  |  |
|  |  |  |  | R.ENPGCLFIATNR.D |  |  |  |
|  |  |  |  | K.LIEGVPETLDMLR.A |  |  |  |
|  |  |  |  | K.LIEGVPETLDMLR.A |  |  |  |
|  |  |  |  | K.GDKLIEGVPETLDMLR.A |  |  |  |
|  |  |  |  | K.TLLVLSGVTSISMLESPENK.I |  |  |  |
| 24 | Chloroplast fructose-1,6-bisphosphatase I [Fragaria ananassa] | 1 (3) | 95/53 | R.YIGSLVGDFHR.T | A8VYM0 | 48.7/5.1 | 44.7/5.2 |
|  |  |  |  | R.VLDIQPTEIHQR.V |  |  |  |
|  |  |  |  | K.YIDDLKDPGPSGKPYSAR.Y |  |  |  |
| 25 | ATP synthase subunit beta [Physalis sp. P078] | 9 (3) | 140/53 | R.IVGEEHYETAQKVK.Q | A8Y6H4 | 16.9/5.3 | 36/5.2 |
|  |  |  |  | R.IVGEEHYETAQRVK.Q |  |  |  |
|  |  |  |  | K.GIYPAVDPLDSTSTMLQPR.I |  |  |  |
| 26 | Ribulose bisphosphate carboxylase large chain [Leucas capensis] | 9 (4) | 172/54 | K.DTDILAAFR.V | A2VAR6 | 50.9/6.7 | 49.7/6.6 |
|  |  |  |  | R.FLFCAEAIYK.S |  |  |  |
|  |  |  |  | K.LNYYTPEYETK.D |  |  |  |
|  |  |  |  | K.TFQGPPHGIQVER.D |  |  |  |
| 27 | Dihydrolipoyl dehydrogenase 1, mitochondrial [Arabidopsis thaliana] | 7 (3) | 140/52 | R.TPFTSGLDLEK.I | Q9M5K3 | 56.4/6.8 | 54.2/7.0 |
|  |  |  |  | K.EAAMATYDKPIHI.- |  |  |  |
|  |  |  |  | K.ALLHSSHMYHEAK.H |  |  |  |
| 28 | Carbonic anhydrase, chloroplast [Arabidopsis thaliana] | 33 (8) | 505/43 | K.YMVFACSDSR.V | Q56X90 | 27.6/6.5 | 28.5/5.3 |
|  |  |  |  | R.NIANMVPPFDK.V |  |  |  |
|  |  |  |  | K.YETNPALYGELAK.G |  |  |  |
|  |  |  |  | R.NIANMVPPFDKVK.Y |  |  |  |
|  |  |  |  | K.VENIVVIGHSACGGIK.G |  |  |  |
|  |  |  |  | K.EKYETNPALYGELAK.G |  |  |  |
|  |  |  |  | K.VISELGDSAFEDQCGR.C |  |  |  |
|  |  |  |  | R.EAVNVSLANLLTYPFVR.E |  |  |  |
| 29 | Putative uncharacterized protein [Sclerotinia sclerotiorum] | 17 (2) | 114/54 | R.SASDAFGLISIR.S | A7ES05 | 17.2/4.6 | 19.4/4.5 |
|  |  |  |  | R.SGSNLQNQAINAFQGGLWIGK.E |  |  |  |
| 30 | Elongation factor 1-beta [Sclerotinia sclerotiorum] | 11 (2) | 75/55 | R.SYIVGYSPSQADVAVFK.A | A7EN12 | 34.7/4.7 | 25.5/4.4 |
|  |  |  |  | K.LVAVGFGIK.K |  |  |  |
| 31 | Aspartate protease [Sclerotinia sclerotiorum] | 22 (4) | 289 /53 | R.SGHDIYTSSK.S | A7ECZ2 | 34.8/4.3 | 41.7/5.1 |
|  |  |  |  | K.GASYSNSYGGYVFPCSATLPTLSFK.I |  |  |  |
|  |  |  |  | K.YTGSLTYTSVSSGNGFWEFPSTSYK.V |  |  |  |
|  |  |  |  | R.SLSGYSWDISYADGSGASGVVGTDTVTIGK.T |  |  |  |
| 32 | Putative uncharacterized protein [Sclerotinia sclerotiorum] | 13 (5) | 149/53 | K.ALAPEYEEAATTLKEK.K | A7ECC8 | 58.5/4.9 | 57.9/4.8 |
|  |  |  |  | K.VFRGPDNVSPYSGAR.K |  |  |  |
|  |  |  |  | K.AEGVSFPSIVLYK.S |  |  |  |
|  |  |  |  | R.TSLAEALKPIAEKHR.G |  |  |  |
|  |  |  |  | K.FVQQYVDGKVEPSIK.S |  |  |  |
| 33 | Putative uncharacterized protein [Sclerotinia sclerotiorum] | 52 (4) | 148/54 | R.EGKPVSHAFAK.E | A7E835 | 17.1/5.1 | 14.6/5.0 |
|  |  |  |  | K.ELLAGFAAGEVDKLVETK.G |  |  |  |
|  |  |  |  | R.SQEQAEHLYDQHYGQDDQYDPNQR.D |  |  |  |
|  |  |  |  | R.DAPEHFNRYDNNW.- |  |  |  |
| 34 | ATP synthase subunit beta [Sclerotinia sclerotiorum] | 14 (6) | 294/53 | K.IGLFGGAGVGK.T | A7ER40 | 49.6/5.2 | 55.6/5.2 |
|  |  |  |  | K.VVDLLAPYAR.G |  |  |  |
|  |  |  |  | R.VQQMLQEYK.S |  |  |  |
|  |  |  |  | R.VVGQDHYDTATR.V |  |  |  |
|  |  |  |  | K.AHGGYSVFTGVGER.T |  |  |  |
|  |  |  |  | R.GISELGIYPAVDPLDSK.S |  |  |  |
| 35 | Malate dehydrogenase [Sclerotinia sclerotiorum] | 17 (2) | 114/54 | R.SASDAFGLISIR.S | A7ES05 | 35.2/6.9 | 19.4/4.5 |
|  |  |  |  | R.SGSNLQNQAINAFQGGLWIGK.E |  |  |  |
| 36 | Glyceraldehyde 3-phosphate dehydrogenase [Botryotinia fuckeliana] | 9 (3) | 125/53 | K.YDSTHGQFK.G | A6SGS7 | 42.1/6.6 | 36.7/5.9 |
|  |  |  |  | R.VLDLLHYISK.V |  |  |  |
|  |  |  |  | K.LVSWYDNEWGYSR.R |  |  |  |
| 37 | Putative uncharacterized protein [Sclerotinia sclerotiorum] | 11 (3) | 123/54 | K.TIHWINEAGK.A | A7F824 | 43.1/6.1 | 41.3/5.6 |
|  |  |  |  | K.LIYGDGSGDTFK.S |  |  |  |
|  |  |  |  | K.ELITEADQDGGIGTYNTQDR.V |  |  |  |

^A^ Spot numbers as given on the 2-D gel image (Figure 3)

^B^%SC/(NP) =Percent sequence coverage/number of peptide(s) matched

^C^ Score: Mascot threshold score for each identified protein as described in materials and methods

^D^The sequence of the matched peptide(s)

^E^ Accession number for proteins generated by the MASCOT search

^F^Exp MW/pI (KDa/pI): Experimentally determined (from gels) molecular weight and isoelectric point of differential proteins.

^G^Theo MW/pI (KDa/pI): Theoretical molecular weight and pI values.
